# Management of Hip Instability in Spinal Muscular Atrophy in the Disease-Modifying Therapy Era: The “Late Autumn” or “Early Spring” of Neuro-Orthopedics?

**DOI:** 10.3390/biomedicines14102138

**Published:** 2026-09-22

**Authors:** Vladimir Kenis, Alexey Kurenkov

**Affiliations:** 1 H. Turner National Medical Research Center for Children’s Orthopedics and Trauma Surgery, Parkovaya 64–68, Pushkin, 196603 Saint Petersburg, Russia; 2 North-Western State Medical University Named After I.I. Mechnikov, 191015 Saint Petersburg, Russia; 3 National Medical Research Center of Children’s Health, Lomonosovsky Avenue, 2 Building 1, 119991 Moscow, Russia

**Keywords:** neuro-orthopedics, hip joint, subluxation, dislocation, spinal muscular atrophy, diagnostics, surveillance, surgery

## Abstract

Neuro-orthopedics is a field comprising knowledge and methods aimed at the diagnosis and treatment of secondary musculoskeletal disorders resulting from primary nervous system pathology. Historically, the greatest step in its development was associated with overcoming the consequences of the polio epidemic, which marked the “early spring” of pediatric orthopedics. However, for progressive neuromuscular diseases like spinal muscular atrophy (SMA), extrapolation of principles developed for poliomyelitis and cerebral palsy did not produce the desired results, leading to a “late autumn” stage, where aggressive surgical intervention was deemed inappropriate, with surgery only recommended as a palliative for intractable pain or care issues. The advent of disease-modifying therapy for SMA opened a new era. Although patients now survive longer, those with SMA type 1 often do not achieve independent standing or walking, predisposing them to hip subluxation and dislocation. Hip joint pathology in neuro-orthopedic diseases remains a significant clinical challenge; however, no unified terminology exists, and neurogenic hip dislocation differs from DDH in being acquired and progressive, requiring dynamic surveillance rather than a one-time assessment. Comparison of current practices across conditions (DDH, CP, myelomeningocele, arthrogryposis, and SMA) reveals heterogeneous diagnostic criteria, screening protocols, and treatment goals. The migration percentage, used in CP, may be an important criterion, but the threshold may differ. Despite a decade of experience with targeted therapy, no significant prospective comparative clinical study has evaluated reconstructive hip surgery outcomes in SMA. Thus, direct application of principles from other conditions is unlikely to resolve the uncertainty. Residual motor function, as in MMC, the migration percentage, contractures, and upper-limb function should be considered. The challenge parallels the “early spring” era, when thousands of polio survivors needed care. We must design fundamentally new frameworks for solving secondary problems after patients have received a chance at life. International, interdisciplinary, integrative research is urgently needed to define diagnosis, screening, prevention, and surgical indications for hip instability in SMA in the DMT era.

## 1. Introduction

Neuro-orthopedics encompasses the diagnosis and treatment of secondary musculoskeletal disorders arising from primary nervous system pathology [1,2]. The most studied neuro-orthopedic conditions include cerebral palsy, the sequelae of neuroinfections (polio, encephalitis, peripheral neuropathies), and myelomeningocele (MMC) [3,4]. Arthrogryposis is closely related to this field [5]. Neuromuscular diseases (SMA, hereditary polyneuropathies, muscular dystrophies, and myopathies) represent an increasingly important and expanding group in terms of prospects [6].

As with any other traditional field of medicine, the history of neuro-orthopedics is difficult to trace to its formal origins. However, it is certain that, regardless of who is considered the founder of this discipline, the greatest step in its historical development was associated with overcoming the consequences of the polio epidemic—a catastrophe that swept the planet in the first half of the 20th century, causing suffering in hundreds of thousands of children. This need allowed neuro-orthopedics to evolve from the stage of describing individual cases and methods to an organized system of diagnosis, treatment, and rehabilitation [7,8]. The “early spring” of pediatric orthopedics was the search for ways to treat patients with the consequences of polio. At this stage of development, when anesthesia, antibiotics, and modern rehabilitation methods were not widely available, several generations of outstanding orthopedists created a new field of science that required not only mastery of surgical techniques, but also a deep understanding of neurology, physiology, and biomechanics [9]. After the Second World War, when the value of every human life became especially evident, the rapid development of neonatology, together with the previous achievements of orthopedics in the treatment of poliomyelitis, gave rise to another—today the major—area of neuro-orthopedics—the treatment of children with cerebral palsy [10]. It was the study of the biomechanics of gait in children with cerebral palsy that gave impetus to the development of modern technologies for three-dimensional motion analysis [11]. Somewhat later, methods for treating children with MMC and arthrogryposis began to develop as a systemic approach [12,13]. The treatment of neuromuscular diseases lagged somewhat behind the aforementioned diseases because many achievements of orthopedic treatment were undermined by steady progression (for some diseases—with an inevitably fatal outcome). An attempt to extrapolate to these diseases (especially SMA and Duchenne muscular dystrophy—the most common form of progressive neuromuscular disease with severe orthopedic impairments) using the mechanistic principles developed in patients with poliomyelitis and cerebral palsy did not produce the desired results. Consequently, most national and international guidelines did not consider orthopedic surgery a method for improving motor function in children with these diseases [14,15]. Figuratively speaking, neuro-orthopedics at this point in its development had reached the “late autumn” stage, in which, owing to the accumulated achievements and mistakes of previous generations of orthopedists, disappointing but pragmatic conclusions were drawn about the inappropriateness of aggressive surgical intervention in these patients.

Recent advances in genetics, coupled with biotechnology, have led to incredible breakthroughs in this field, leading to the emergence of disease-modifying therapy for a number of previously fatal diseases [16]. Thanks to this, the professional orthopedic community is now under close scrutiny from geneticists, neurologists, physical therapists, and, naturally, parents and patients.

Surgical treatment of hip joint pathology in neuro-orthopedic diseases is one of the most significant clinical applications, which is the subject of extensive debate between orthopedic surgeons, neurologists, and physical medicine specialists [17]. The hip joint is the cornerstone of locomotion. Its pathology leads to significant functional impairments and can markedly limit the effectiveness of other treatment methods. Repeated attempts have been made to introduce a more unified term (neurogenic instability of the hip, paralytic instability of the hip, spastic disease of the hip, etc.) [18]. However, a single designation for a condition that is generally fairly understandable and relatively similar across different diseases still does not exist. Naturally, hip joint pathology associated with neurological diseases is compared to a dysplastic hip joint (immaturity/dysplasia/subluxation/dislocation), although the terminology is also far from universal.

The aim of this narrative review was to analyze current practices of diagnostics, screening, prevention and management of the best-studied pediatric orthopedic conditions with predominant hip involvement with a focus on neurogenic disorders. We conducted the literature search and selection intentionally, aiming to illustrate the discussed problem and formulate a framework for future research.

We searched PubMed and Google Scholar using combinations of the following terms: (“spinal muscular atrophy” OR “SMA”) AND (“hip” OR “hip dislocation” OR “hip subluxation” OR “migration percentage”); and for comparator conditions: (“developmental dysplasia of the hip” OR “cerebral palsy” OR “myelomeningocele” OR “arthrogryposis”) AND (“hip” OR “subluxation” OR “dislocation” OR “surveillance”). We screened titles and abstracts and selected articles by expert consensus. Because this was a purposive narrative review, we did not aim for exhaustive retrieval; more than 350 records were identified and 75 were included as key references. The selection of the literature was intentionally based on the expert consensus of both authors. The selection process was, by its nature, partly subjective, especially because the literature on the comparator conditions is very large and not always directly related to SMA. We did not impose strict chronological limits, since many older publications on DDH, CP, MMC, and arthrogryposis remain highly relevant and practically important. We acknowledge that this approach carries a potential selection bias, and we have made this limitation transparent.

## 2. Comparative Analysis of Current Practices Across Conditions

According to the search strategy described above, we arranged the literature according to the following framework: “diagnostic standard—radiographic diagnostic criteria—screening—prevention—non-surgical management—primary indications for surgery management—secondary indications for surgery—surgical management (reconstruction)—palliative surgery”. Within this framework we selected the data regarding the most studied neurogenic conditions (CP and MMC). Historically, attempts were made to attribute the etiology of DDH to occult neurological impairment [19]. There is currently no evidence to support such conclusions [20]. DDH is not a neuromuscular disease and we used it only as a comparator—a reference model of primary hip instability without a neurological background. Its inclusion is intended to contrast the well-developed diagnostic, screening, and treatment pathways for DDH with the much less defined situation in neurogenic and neuromuscular hip instability. Arthrogryposis (mostly amyoplasia) was also used more as a comparative model of severe contractures of hypothetical spinal origin [21] than as a pure neuromuscular condition. In our opinion, this outline of the conditions can better describe the landscape of pediatric hip disorders for the elaboration of the possible multidimensional framework for the optimal studying of hip problems in SMA.

The analysis of current practices in the treatment of hip instability in children with various diseases can help to identify promising areas for addressing the problems of diagnosis, screening, prevention, and treatment of hip instability in children with neuromuscular diseases receiving targeted therapy, using SMA as an example.

## 3. Developmental Dysplasia of the Hip

DDH is not a neuromuscular disease. It is included here only as a reference model of primary hip instability without a neurological background. In DDH, early diagnosis with hip ultrasound is of the utmost importance [22]. At later stages, for the detection of residual dislocations or dysplasia, radiographic examination with the assessment of the most well-proven radiographic parameters (acetabular index, Wiberg center-edge angle, neck-shaft angle, Sharp angle and a large number of less commonly used parameters) is the standard. Accordingly, screening for hip dysplasia in children in the first months of life is a widespread practice and a well-documented method for minimizing (and ideally eliminating) late diagnosis and untimely treatment. In a number of countries, screening is universal, but the possibilities of selective screening are also widely discussed, although the optimal selection strategies have not yet been definitively determined.

For DDH, prophylactic positioning (swaddling, use of slings and transportation devices) and avoiding unfavorable positions (tight swaddling and traditional cradles with excessive hip adduction) have been considered preventive measures. Accordingly, wide swaddling, so-called “healthy swaddling,” can be considered a preventive measure. Its effectiveness as a population-based measure has not yet been fully proven, but it shows promising preliminary results.

The goal of treatment (both conservative and surgical) in DDH is to restore function and anatomy as fully as possible. Surgical treatment methods for DDH are well-documented in the pediatric orthopedic literature and do not require detailed discussion within the scope of this publication. Ideally, the vast majority of dislocations should be detected promptly and successfully treated through conservative treatment using external devices (in most practices, a Pavlik harness). Irreducible dislocations are managed by either close reduction with cast immobilization or open reduction. The use of bony procedures at this stage is optional. Residual cases require surgical treatment, the extent of which depends on the anatomical abnormalities, age, and practices in the clinic/country/school (open reduction, femoral osteotomy, pelvic osteotomy, tendoplasty, capsuloplasty in various combinations) [23]. Palliative surgeries (such as pelvic support, Chiari procedure, resections, and arthrodesis) are currently rarely considered for DDH because of the development of reconstructive techniques and the proven effectiveness of early arthroplasty in selected cases.

## 4. Cerebral Palsy

In cerebral palsy, because of the acquired progressive nature of the hip joint pathology, screening trends are different and are described in the terminology of a “surveillance program”. In fact, we are also talking about universal (at least for certain age groups) or selective screening of the population of children with cerebral palsy. The selection criteria, which are discussed in the literature, in accordance with the nature of the pathology, include age, GMFCS level, and the presence of contractures. Because of the possibility of an inaccurate assessment of these parameters (the difference between chronological age and biological maturity and the less-than-ideal inter-rater reliability of contracture assessment and motor tests), universal surveillance programs for cerebral palsy seem optimal today [24].

In contrast to DDH, in cerebral palsy, anatomical indicators in most studies do not have independent significance as monitoring parameters, giving way to the migration percentage. The effectiveness of positioning and prevention of progression of hip subluxation in cerebral palsy is controversial. Among the preventive measures that have been considered are positioning, passive standing, the use of abduction splints of various designs adapted for children with cerebral palsy, botulinum toxin therapy, chemical denervation, and prophylactic surgical interventions (tenotomies, neurotomies). Despite the significant number of such studies, they share common limitations: difficulties in selecting ideal comparison groups, lack of long-term studies, and the ethical implications of comparative research in this area. The most common empirical approach to prevention in children with cerebral palsy is the recommendation to avoid “forbidden postures”—severe asymmetry, prolonged hip flexion and adduction, and unjustified reductions in weight-bearing. However, the scientific evidence for these measures is rather limited, and the recommendations are mostly situational.

In CP, the goal of treatment is entirely different from DDH. Since basic function, within the generally accepted GMFCS framework, is at best not improved, the goal of treatment in ambulatory children (GMFCS 1–3) is to preserve function and improve quality of life (prevent early osteoarthritis in the future). In more severe conditions (GMFCS 4–5), the goal of treatment is to prevent or reduce pain, optimize positioning, and facilitate daily care.

The question of a proactive versus reactive strategy in CP remains a subject of debate. In most cases, a reactive tactic is recommended in accordance with the established critical threshold value of the migration percentage (in most publications, 40%) [25]. Studies regularly appear that cast doubt on the universality of this principle. There is evidence of decreased effectiveness of surgeries performed with high migration percentage values in older children (however, as a rule, in the medium term), as well as studies demonstrating less-than-ideal results of surgeries performed at a younger age. Thus, the experience, personal knowledge, and intuition of the physician continue to play a significant role in the choice of treatment strategy for hip instability in cerebral palsy. The role of palliative surgery in cerebral palsy is widely discussed as an alternative to reconstructive surgery in patients with the most severe motor impairments, with various types of resection arthroplasty being considered. Hip arthroplasty in cerebral palsy is a highly debated and rapidly developing area of research; however, further long-term observation of patients undergoing surgery is required for more definitive conclusions regarding indications for surgery, its technical nuances, and so on.

## 5. Myelomeningocele and Arthrogryposis

In MMC, hip joint examination at a young age is not performed as a screening procedure. Examinations are usually performed based on clinical indications, such as contractures, clinically evident instability, or incidental findings during spinal or urinary tract imaging that raise suspicion of hip pathology [26]. In arthrogryposis, examination is similarly indicated by the physician based on clinical data, the presence of contractures, and the patient’s motor status [27].

Because of the lack of a standard monitoring protocol in MMC, different studies have evaluated different parameters. Both anatomical indices (acetabular index, CEA, and, to a lesser extent, the neck-shaft angle, femoral torsion, and Sharp angle) and the dynamic migration percentage (MP) have been mentioned. Hip dislocations in arthrogryposis are mainly congenital and can be classified as teratologic.

The treatment goal for MMC also depends on the functional status, which is determined by the neurological level: in patients with high segmental levels (thoracic and upper lumbar), surgical treatment is not indicated in most cases, since hip dislocation does not interfere with positioning and care and does not lead to pain (because of insensitivity). In exceptional cases (presence of pain, significantly limited positioning and care), the possibility of surgical treatment for selected indications is discussed with patients and caregivers [28]. For MMC, the situation is even less clear. The determining factor for choosing a treatment strategy is generally considered to be not radiographic impairment but motor potential (more precisely, the neurosegmental level) [29]. Because of the accompanying hypoesthesia/anesthesia in most cases, pain is rarely a significant complaint in children with MMC, even with significant joint abnormalities. It has been shown that at the upper thoracic level, reconstruction of the hip joints in most cases indirectly led to secondary deterioration (because of the resulting “cascade of fractures”) [30], and an anatomically good result of surgical correction did not correlate with quality of life and motor function. In cases where the operation was aimed at preserving function (mid-lumbar, lower lumbar, sacral levels), the choice of surgical treatment method was based on anatomical parameters, similar to those in DDH, with the addition in some cases of tendon and capsule procedures, the role of which in the overall result is not however well defined [31]. Arthroplasty in children with MMC is rarely the subject of individual studies, but overall, when motor function is sufficiently preserved, the indications and techniques appear broadly comparable to those for DDH.

Reconstructive surgery of the hip joint in arthrogryposis is extensively presented in the literature, but in a fragmented manner [32,33]. Historically, the earliest systematic approach to this problem was reflected in the works of Staheli. Based on the low motor potential of patients and the presence of concomitant deformities that also require treatment, the outstanding pediatric orthopedist of our time recommended not striving for anatomical restoration but rather preserving function [34]. In this context, preference was given to open reduction in the early stages [35]. Returning to the criteria for evaluation and monitoring, Staheli considered a migration percentage of up to 50% an acceptable treatment result, sufficient for achieving functional goals (in contrast to, for example, cerebral palsy).

For MMC and arthrogryposis, there are currently no clear recommendations for the prevention of hip instability. General recommendations based on traditional practice mainly concern maintaining symmetry. Attempts at preventive surgical interventions aimed at restoring balance (tenotomy of the adductors and iliopsoas muscles, various types of abductor reinforcement) have not demonstrated consistent, reproducible effectiveness in large cohorts.

## 6. Spinal Muscular Atrophy

In the pre-DMT era, most guidelines and publications recommended regular radiographic examination of the hip joints in SMA. However, the frequency of mandatory examinations and their relationship to age and motor status are not supported by the literature. Also, because of the lack of an established monitoring regimen, different researchers have used different indices, as in the previous two conditions, which included both dynamic and anatomical parameters [36]. However, the lack of a clear relationship between baseline parameters and their dynamics as a criterion for deterioration precludes both the definition of a point of no return and any meaningful discussion of indications for intervention. Data on SMA also do not provide clear guidance on hip subluxation prevention. General recommendations emphasize the advisability of correct positioning [37]. However, excessive hip abduction as a possible means of preventing subluxation progression in the early stages requires more convincing justification than just theory, since abduction contractures in neuromuscular diseases (for example, in Duchenne muscular dystrophy) are no less a problem than hip dislocation.

Indications for surgical treatment in SMA in the pre-DMT era were even less clear. Publications mention, in particular, intractable pain and contractures, but leave complete uncertainty regarding age, motor impairment, and radiographic parameters sufficient for a surgical decision [38].

Surgical treatment methods used for hip instability in children with SMA in the pre-DMT era (presumably before recommendations against surgical treatment were issued) also generally fit within the paradigm of reconstructive surgery for neurogenic hip instability [39]. Historically, open reductions, femoral osteotomies, pelvic osteotomies, and various soft-tissue procedures were cited, but none demonstrated superiority over any method because of the overall negative treatment outcome, and none of these interventions resulted in the expected improvements in function and quality of life.

Future studies should prioritize patient-centered outcomes, including pain, sitting balance, positioning, ease of care, preservation of standing or ambulatory potential, quality of life, and caregiver burden [40]. Anatomical correction alone may not justify extensive reconstructive surgery in SMA and uniform standards for hip instability in SMA cannot yet be established at the current level of evidence.

## 7. Discussion

DDH is one of the fundamental issues in general pediatric orthopedics. Over more than a century of scientific study, diagnostics (including screening) [41], prevention (e.g., wide swaddling) [42], conservative and surgical treatments have been well-developed for this condition. The optimal treatment outcome is to achieve a virtually complete recovery. Attempts to extrapolate mechanistic principles in the diagnosis and treatment of DDH to neurogenic conditions have demonstrated the impossibility of straightforward application to these diseases.

Neurogenic hip dislocation is not congenital in most cases (at least in cerebral palsy, in most cases of MMC, and in progressive neuromuscular diseases like SMA). Most bony deformities (femoral and pelvic) develop and progress postnatally—for example, in the case of Charcot–Marie–Tooth hereditary neuropathy, this may even occur in adolescence [43]. That is, screening strategies for neurogenic instability should not be a one-time process (pathology present/pathology absent), but should be dynamic, spanning at least several years [44].

The next important difference, arising from the previous one, is the uncertainty of the timing of intervention. Since changes develop gradually over several years, a decision threshold regarding the need for surgery is necessary. If the pathology is discovered incidentally at an advanced stage, the choice is relatively obvious. However, if the patient undergoes regular screening as part of a surveillance program, the timing of intervention must be carefully selected based on rather arbitrary criteria (for example, the migration percentage for cerebral palsy), and this should involve shared decision-making with non-surgical specialists and the parents [45].

Another complex issue that should be taken into account is the interplay between primary neurological impairment and secondary functional limitations. Even in cerebral palsy, which is traditionally considered a non-progressive disease from a neurological point of view, orthopedic manifestations are in fact progressive (onset and progression of contractures and deformities) [46]. Thus, one-time resolution of hip joint pathology, even through an effective operation, does not mean that this issue is resolved forever [47]. On the contrary, recent studies have called for these potential complications to be taken into account [48], although it is not yet entirely clear how. Other factors that may influence treatment choice and outcome include osteoporosis, selective motor control, spasticity or hypotonia, upper-limb function, and cognitive impairment. Ultimately, any seemingly insignificant factor can compromise the results of even a perfectly performed operation.

Returning to neuromuscular diseases, the main limiting factor, as already mentioned, is the progressive nature of the disease itself. In this case, a natural question arises: is it necessary to perform extensive operations if deterioration or loss of motor function is inevitable (sometimes within a fairly short predictable timeframe). Until now, this thesis has dominated the discussion of such cases. In this regard, the general conclusion of both empirical studies [49,50], as well as the consensus decisions [51], was the position shared by the majority of orthopedic surgeons that hip surgery in children with progressive neuromuscular diseases should be performed only as a palliative in the presence of persistent pain or intractable problems with positioning and, in general, daily living, care, and hygiene. This conclusion, which is found in the majority of studies (although few in number), also leaves a substantial subjective component for the surgeon and the decision of the parents in determining the indications for surgery.

Thus, the poetic term we used, “the late autumn of neuro-orthopedics”, applies to hip surgery for progressive neuromuscular diseases in approximately the following way: since we cannot preserve or improve function, hip surgery itself is not a determining factor in these patients and can cause negative consequences and complications. Observational strategy and the solution of minor problems (pain, care) are possible in individual cases, and parents and rehabilitation specialists should understand that deterioration is inevitable and the situation is essentially unresolvable.

All of this could be considered a sad axiom were it not for the breakthrough in the treatment of neuromuscular diseases that occurred a decade ago. In 2016, we entered a new era of practical, widespread use of targeted therapy (initially, SMA) [52].

Currently, in real clinical practice for patients with SMA, three main disease-modifying therapy approaches are used: intrathecal administration of an antisense oligonucleotide (nusinersen), an oral small-molecule drug (risdiplam), and a single intravenous infusion of a gene therapy drug (onasemnogene abeparvovec). Randomized clinical trials, although mostly short-term, have been conducted for each of these drugs, and practical experience has accumulated. Analysis of the SMArtCARE registry (84 centers in Germany, Austria, and Switzerland) showed that the majority of patients with SMA (63.8%) remained on the drug with which they started treatment, indicating high satisfaction with the efficacy and tolerability of therapy. Neonatal screening for SMA has led to early detection of the disease, enabling diagnosis in the first weeks of life and the earliest possible start of disease-modifying therapy. By 2024, the majority of newborns identified by screening with 2–3 copies of the SMN2 gene received onasemnogene abeparvovec as a first-line treatment [53]. In terms of the development of secondary neuro-orthopedic complications, primarily hip joint pathology, patients with SMA type 1 still pose the greatest challenge. This is because in a large number of such patients, the onset of the disease is observed in the first month of life, and even despite the early administration of disease-modifying therapy, this does not lead to the restoration of the child’s motor development, especially independent standing and walking [54,55]. The presence of muscle weakness in the proximal muscle groups of the lower extremities in the absence of adequate weight-bearing in such patients creates predisposing factors for the development of subluxation and dislocation of the hip. Considering that patients with SMA type 1 constitute the main cohort (50%) of all patients with 5q SMA, the number of patients with potential hip joint pathology will increase.

In practice, the challenge we now face is comparable to that faced by orthopedists in the past during the “early spring” of neuro-orthopedics, when thousands of children with the consequences of polio began to survive. While before the advent of therapy, the number of patients with SMA remained relatively small and constant because of the natural “shift in mortality”—the natural fatal course of the disease—we are now faced not only with increased demand for professional solutions to this unprecedented challenge, but also with an expected (and, in fact, already observed) significant increase in the number of surviving patients. The same will soon apply to Duchenne muscular dystrophy and probably also to hereditary polyneuropathies and a spectrum of similar disorders.

It is becoming increasingly clear that the DMT-treated SMA population is heterogeneous. Motor outcomes may differ substantially according to SMA phenotype, SMN2 copy number, age and functional status at treatment initiation, presymptomatic versus symptomatic treatment, and the specific therapeutic strategy. Future hip surveillance and treatment strategies may need to be stratified according to motor phenotype or functional trajectory rather than conventional SMA type alone. However, because the possible combinations of genetic, clinical, therapeutic, and modifying factors are numerous and still poorly studied, we present this as an important question for future research rather than as a currently actionable algorithm. The concepts of residual motor function, migration percentage, contractures, and upper-limb function are the most promising candidate factors for future investigation. Nevertheless, the idea that disease-modifying therapy may convert a progressive disease into a “residual” state is now clearly described as a hypothesis.

What can modern neuro-orthopedics offer to patients with hip joint pathology associated with neuromuscular diseases, such as SMA? A review of the literature shows that despite a decade of accumulated experience in observing patients who have received therapy, no significant progress has been made in this area to date. We can see isolated anecdotal reports of patients who have undergone surgery (usually not as part of a systematic scientific analysis of surgical results, but as part of complex treatment measures, which makes it difficult to isolate the effect of surgery in the overall outcome). Surprisingly, over these 10 years, no substantial prospective comparative clinical study assessing the results of reconstructive surgery of the hip joint in SMA has been presented. Isolated publications, references, and presentations of clinical cases do not allow definitive judgment.

This article is a narrative, conceptually oriented review. It does not aim to provide new primary data, a systematic review, or a replacement for the existing Delphi consensus on hip management in SMA. Delphi consensus procedures and similar approaches provide valuable expert guidance, but they do not substitute for prospective comparative clinical evidence [56]. The Delphi consensus is an important contribution to clarifying the position of the professional community. Our manuscript does not attempt to replace it; rather, it asks what evidence is still missing and why a consensus-based approach alone cannot answer the key questions. It is an attempt to convert that uncertainty into testable clinical and research questions. Even the title itself is deliberately formulated as a question: the “late autumn” or “early spring” of neuro-orthopedics? It does not assert a new answer. It signals that the field is at a turning point and that the main task is to pose the right questions rather than to claim premature certainty.

It should also be pointed out that the data on DDH, CP, MMC, arthrogryposis, and the pre-treatment natural history of SMA are based on published evidence, whereas the proposed principles for hip management in SMA patients receiving disease-modifying therapy are largely conceptual and hypothetical. These proposals reflect the authors’ opinion and should be regarded as a framework for future investigation rather than as established clinical criteria.

Even well-established diagnostic thresholds (such as the migration percentage threshold established in CP) should not be directly extrapolated to SMA; such extrapolation is problematic because the pathophysiology, muscle imbalance, functional potential, and natural history of hip displacement differ between the disorders. There is a need to better establish the natural history and longitudinal trajectory of migration percentage in treated SMA, and the rate of progression may be as important as any absolute threshold when considering indications for intervention.

Our views, based on analysis of the literature on this issue, are presented in Table 1. In the table, we present information on existing schemes and approaches to diagnosis, screening, prevention, and treatment of hip pathology in the main conditions discussed, including DDH as a non-neuromuscular comparator, cerebral palsy, MMC, arthrogryposis multiplex, and SMA.

Speaking about the limitations, our article overlaps substantially with numerous previous publications on neurogenic hip disease in general and on spinal muscular atrophy in particular. We do not claim to present fundamentally new content. Nevertheless, we believe that our work has a certain originality: it combines not so much theoretical aspects of neurogenic hip disease as a nosological clinical problem, but rather a synthetic approach, primarily presented in Table 1. This approach serves as a link between expert opinions (the content of the Delphi consensus) and the available data on other neurological conditions associated with hip pathology. We also do not claim a separate or isolated vision of hip pathology in SMA. However, in the framework we propose—diagnosis, screening, prevention, non-surgical treatment, surgical treatment, and palliative surgery—we see an opportunity for further clinical research. This explanation may not fully exhaust the question of novelty, but in our view, it provides sufficient justification and prompted us to share this introduction to the problem with colleagues.

In summary, there are currently insufficient scientific data that would allow for a systematic approach to this problem. What should practicing physicians and scientists do when attempting to understand this situation and plan research? We believe it would be useful to summarize and analyze treatment experience for other, better-studied conditions.

## 8. Conclusions

Where will hip surgery in children with SMA evolve in the era of disease-modifying therapy? Are there any current trends toward formulating principles of diagnosis, screening, prevention, surgical treatment, and other technical aspects? Returning to the imagery, is the “late autumn” of neuro-orthopedics continuing, or can we expect a “new spring”? Unfortunately, the available data do not yet allow us to identify trends. Direct application of principles known for DDH, cerebral palsy, arthrogryposis and MMC obviously cannot help make this choice because of the fundamental differences between SMA and the aforementioned conditions. However, we can draw on fragmented experience in each of these areas and assemble the puzzle. It seems most likely that residual motor function will serve as a starting point for selection, as is the case with another flaccid paralysis—MMC. If we assume that the initiation (or, more realistically, the onset of clinical effect) of therapy marks the transition from a progressive to a residual disease, then the motor function preserved at this point can be considered a baseline, with adjustments for growth and physical development. The migration percentage should certainly become an important additional criterion for choosing a treatment strategy, since other anatomical parameters (acetabular index, neck-shaft angle) may not differ substantially in patients with relatively stable joints and with dislocation because of varying degrees of muscle imbalance. However, the choice of migration percentage values as the “point of no return” may differ from that in CP. The presence of contractures should be considered an important criterion for choosing a treatment strategy in SMA. Flexion contractures in the knee joints in cerebral palsy (GMFCS 3–5) do not interfere with sitting and lying and are not considered a significant factor limiting the choice of hip surgery. In SMA, this factor may be important, since even minimal contractures of the knee joints with severe weakness of the knee extensors will not permit functional improvement in most cases. Upper-limb function should also be a significant factor in the choice. For example, in most cases of MMC this function is not strongly affected, allowing significant compensation for lower limb muscle weakness with the use of mobility aids. However, this factor can be a significant limitation in SMA.

## 9. Future Directions

Finally, accumulating long-term observational experience will allow us to correlate long-term therapy expectations with reality. The emergence of additional prognostic factors (the presence of different SMN2 copy numbers, modifier genes, other biomarkers, residual function) and the development of treatments that can not only halt the process of motor neuron degeneration but also improve basic muscle function will introduce even more variables into this equation.

These candidate factors should be tested prospectively. A multicenter cohort of DMT-treated SMA patients could undergo standardized hip surveillance with annual radiographs, migration percentage, contracture assessment, upper-limb function, and motor milestones. The primary outcome could be progression to a predefined MP threshold or need for surgery; secondary outcomes could include pain, sitting balance, standing ability, quality of life, and caregiver burden. Multivariable analysis could identify which factors predict displacement and treatment response and whether SMA-specific thresholds differ from CP.

Having witnessed the remarkable achievements of fundamental science translated into clinical practice and having seen them become reality, we must design fundamentally new frameworks for solving secondary problems after patients have received a chance at life and improved overall health. Classical developments will undoubtedly form the foundation for future research in this area, but they will not eliminate the high uncertainty, which will have to be overcome through joint efforts in international, interdisciplinary, and integrative research.

## Figures and Tables

**Table 1:** Existing schemes and approaches to diagnosis, screening, prevention, and treatment of hip pathology in DDH and neuromuscular diseases.

Diagnosis	DDH (As a Comparative Condition)	CP	MMC	AMC	SMA (Pre-DMT)
Diagnostic standard	Babies—US (Graf) Then—X-ray [22]	X-ray [18]	X-ray [29]	X-ray [57]	X-ray [15]
Radiographic diagnostic criteria	First line: AI, CEA Second line: NSA, Shenton, Sharp [58,59]	First line: MP Second line: NSA, HSA, AI [36,60]	First line: N/E Second line: MP, NSA, AI [26]	First line: N/E Second line: MP, NSA, AI [61]	First line: N/E Second line: MP, NSA, HSA, AI [36]
Screening	US screening in babies (total, selective) [41]	Hip surveillance programs [18,24,44]	N/E (hip surveillance recommended) [12]	N/E (according to clinical findings) [13]	N/E (hip surveillance recommended) [62]
Prevention	Avoidance of straight-leg fixation, healthy swaddling [42]	Positioning, postural management, splints, tone management [63]	N/E (positioning recommended) [64]	N/E [61]	N/E (positioning recommended with caution) [37]
Non-surgical management	Gold standard (splinting, close reduction) [22]	May be used as an adjunctive treatment [65]	limited role [64]	limited role [66,67]	No brace application has been suggested to prevent or slow progression [37,49]
Primary indications for surgery	Irreducibility, age, AI, CEA [23]	MP, pain [68]	Functional level, asymmetry [26,28]	Asymmetry, contractures [27]	Intractable pain [38]
Secondary indications for surgery	NSA, AT, Shenton, Sharp [69]	Age, GMFCS level, asymmetry [68]	Pain (rare), contractures, age, NSA, Shenton, Sharp [70]	Pain (rare), age, NSA, AT, Shenton, Sharp [34]	Contractures, asymmetry (mentioned without established cut-off) [39]
Surgical management (reconstruction)	Residual cases (combination of open reduction, VDRO, pelvic OT) [71]	Combination of open reduction, VDRO, pelvic OT [72]	Combination of open reduction, VDRO, pelvic OT [29]	Combination of open reduction, VDRO, pelvic OT [32,35]	Not recommended [15,50]
Palliative surgery	Not recommended (early THA if necessary) [73]	For residual cases with severe pain and secondary deformity (GMFCS 4–5) [74]	For residual low-functional cases with severe pain and asymmetry [75]	Total hip arthroplasty can be effective for painful joint degeneration in older patients but does not improve ambulatory function [33,66]	For residual cases with severe pain [40]

SMA—spinal muscular atrophy; DMT—disease-modifying therapy; DDH—developmental dysplasia of the hip; CP—cerebral palsy; MMC—myelomeningocele; AMC—arthrogryposis multiplex congenita; AI—acetabular index; CEA—center-edge angle; MP—migration percentage; NSA—neck-shaft angle; HSA—head-shaft angle; GMFCS—Gross Motor Function Classification System; VDRO—varus-derotation osteotomy; OT—osteotomy; THA—total hip arthroplasty; US—ultrasound; N/E—not established.

## Data Availability

No new data were created or analyzed in this study. Data sharing is not applicable to this article.

## Abbreviations

The following abbreviations are used in this manuscript:

SMA	Spinal Muscular Atrophy
DMT	Disease-Modifying Therapy
CP	Cerebral Palsy
MMC	Myelomeningocele
AMC	Arthrogryposis Multiplex Congenita
DDH	Developmental Dysplasia of the Hip
AI	Acetabular Index
CEA	Center-Edge Angle
MP	Migration Percentage
NSA	Neck-Shaft Angle
HSA	Head-Shaft Angle
GMFCS	Gross Motor Function Classification System
VDRO	Varus-Derotation Osteotomy
OT	Osteotomy
THA	Total Hip Arthroplasty
US	Ultrasound
